# Supplementary figures and images for: A multi-hierarchical approach reveals d-serine as a hidden substrate of sodium-coupled monocarboxylate transporters
Source: eLife. 2024 Apr 23;12:RP92615. doi: 10.7554/eLife.92615 (PMC11037918; doi:10.7554/eLife.92615)

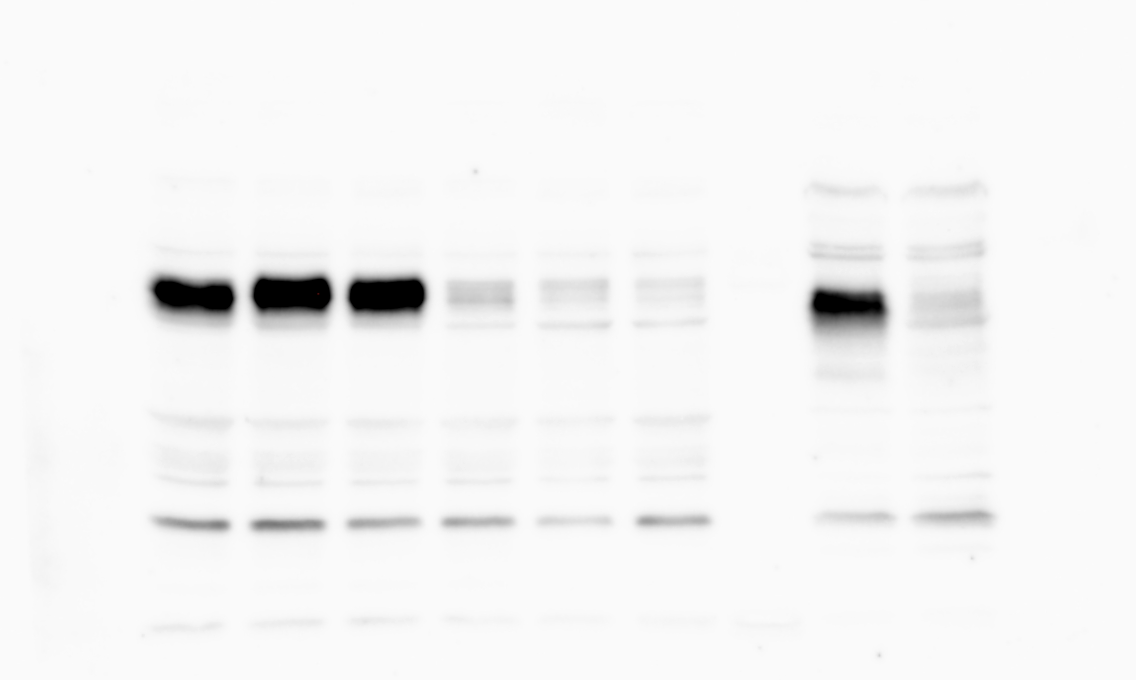

Supplement: Figure 3—source data 1. [file elife-92615-fig3-data1.zip › Figure 3 - source data 1 (orginal WB).tif]

Figure 3

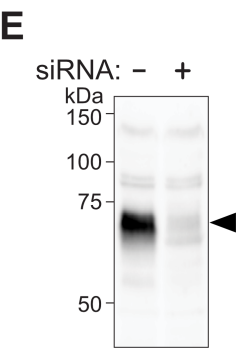

Source data

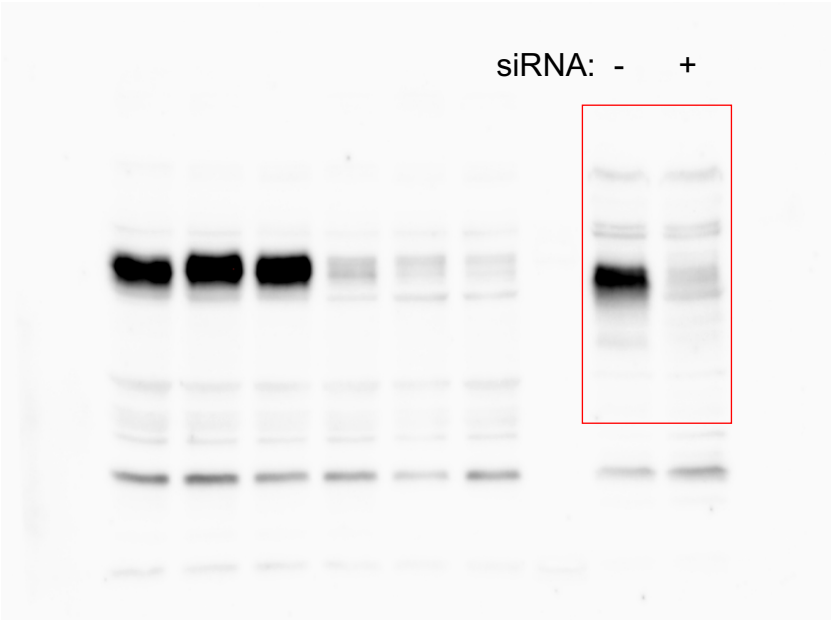

Supplement: Figure 3—source data 2. [file elife-92615-fig3-data2.pdf]

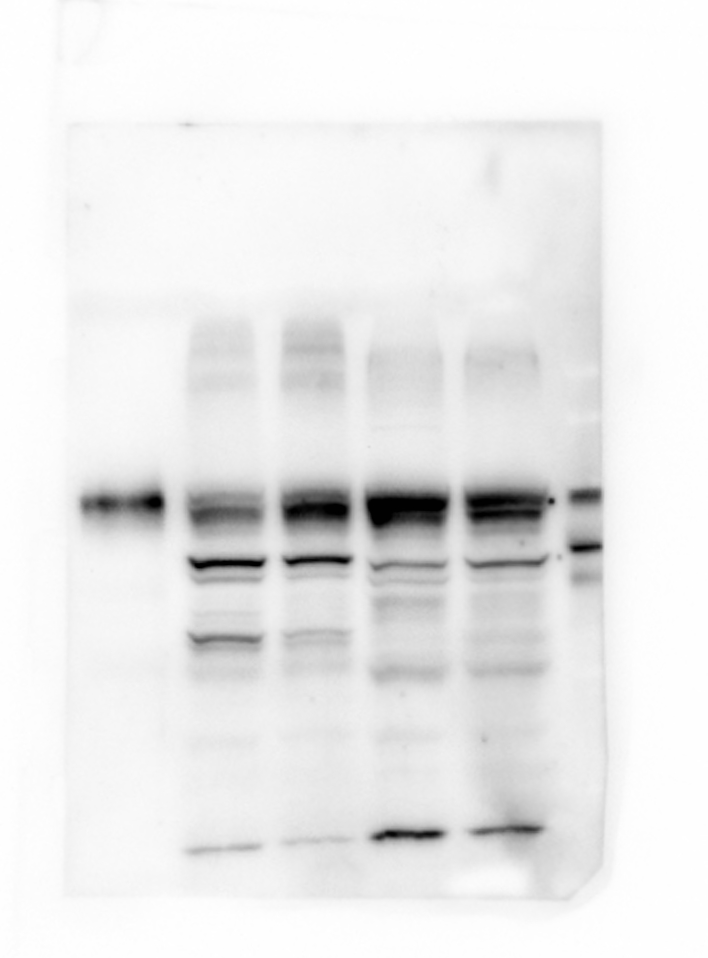

Supplement: Figure 3—figure supplement 1—source data 1. [file elife-92615-fig3-figsupp1-data1.zip › Figure 3-figure supplement 1 - source data 1 (original WB for NT).tif]

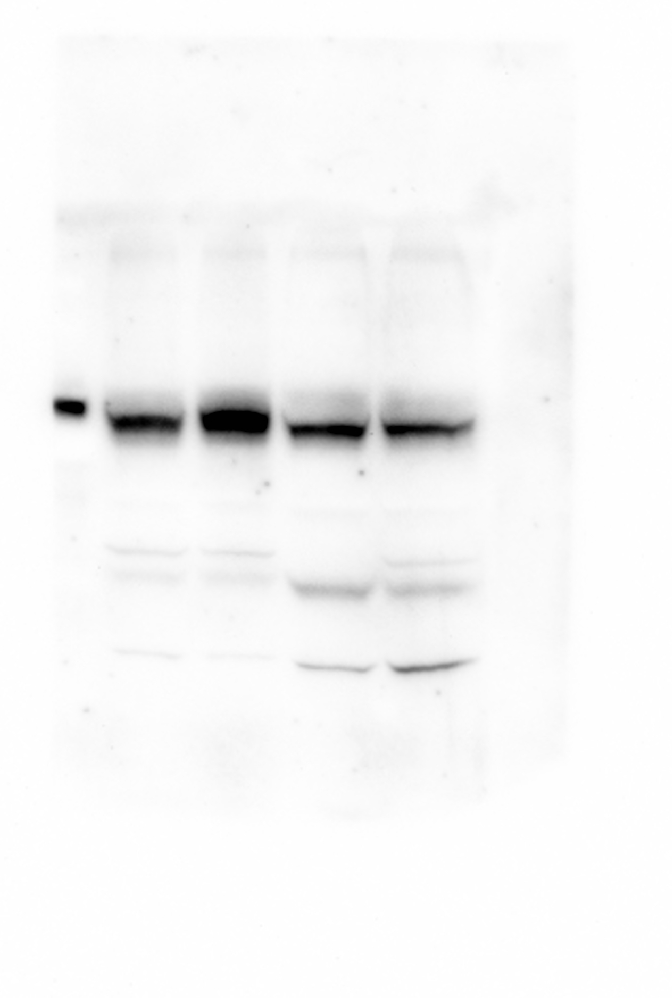

Supplement: Figure 3—figure supplement 1—source data 2. [file elife-92615-fig3-figsupp1-data2.zip › Figure 3-figure supplement 1 - source data 2 (original WB for CT).tif]

Figure 3-figure supplement 1

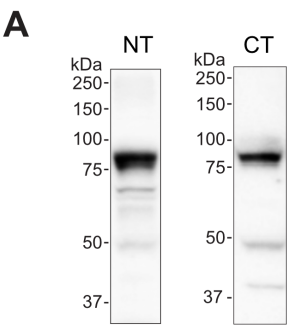

Source data

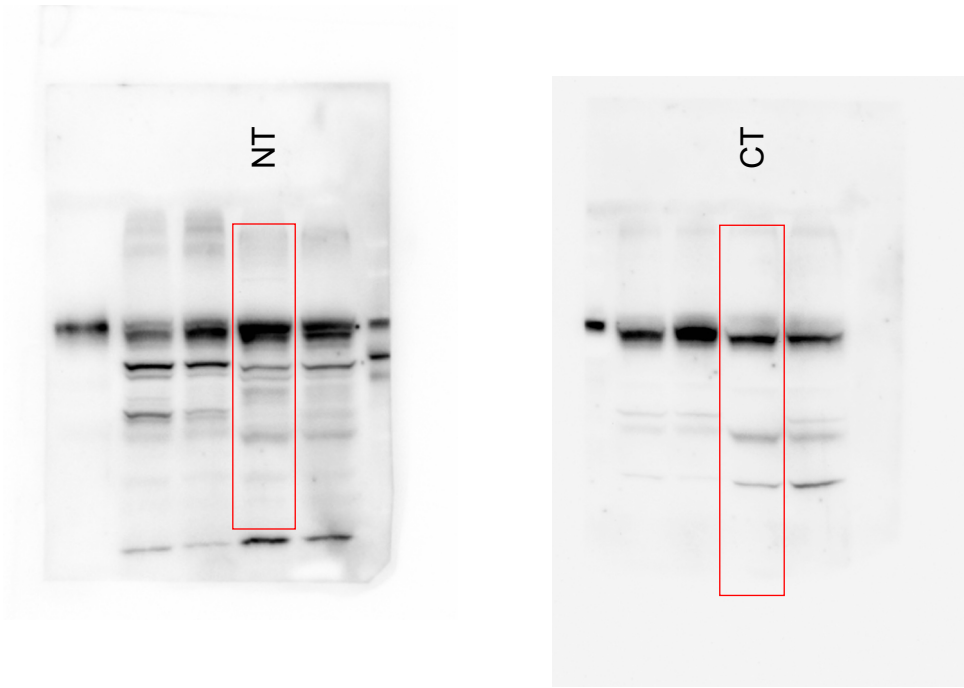

Supplement: Figure 3—figure supplement 1—source data 3. [file elife-92615-fig3-figsupp1-data3.pdf]

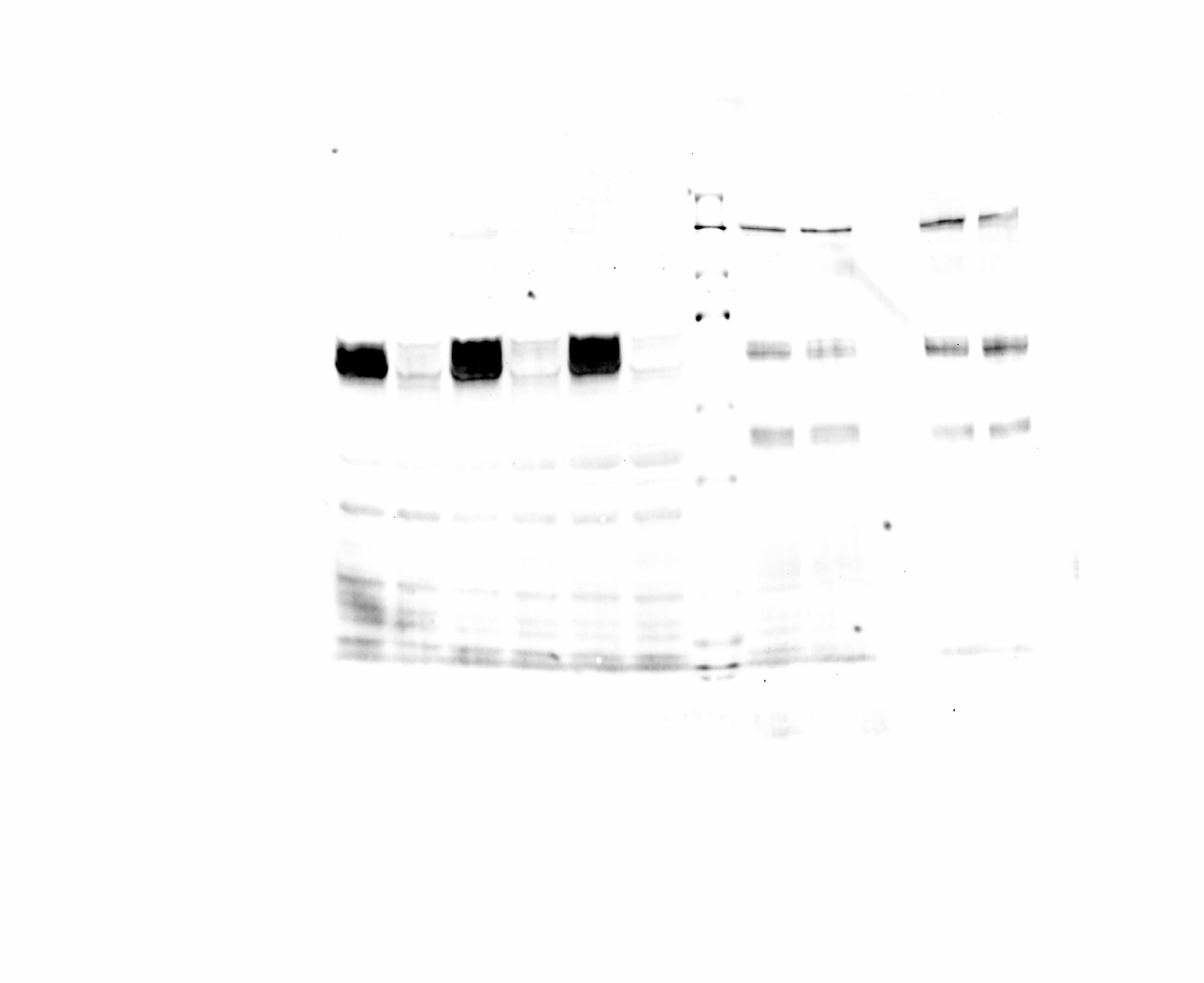

Supplement: Figure 5—figure supplement 1—source data 1. [file elife-92615-fig5-figsupp1-data1.zip › Figure 5-figure supplement 1 - source data 1 (original WB-anti ASCT2).tif]

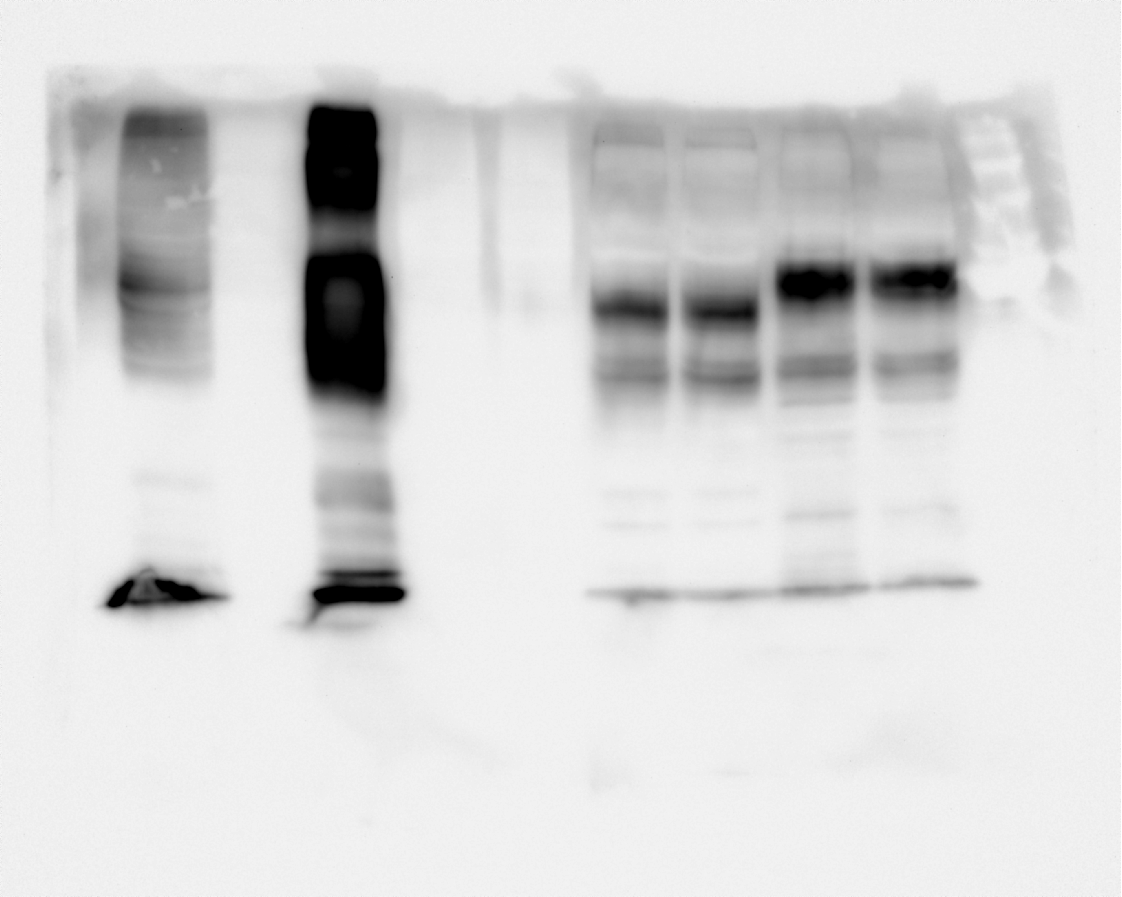

Supplement: Figure 5—figure supplement 1—source data 2. [file elife-92615-fig5-figsupp1-data2.zip › Figure 5-figure supplement 1 - source data 2 (original WB-anti FLAG).tif]

Figure 5-figure supplement 1

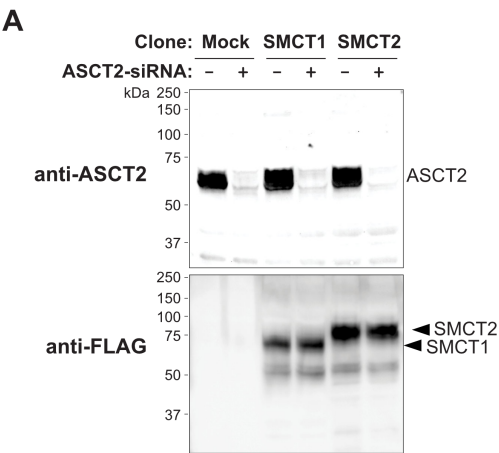

Source data

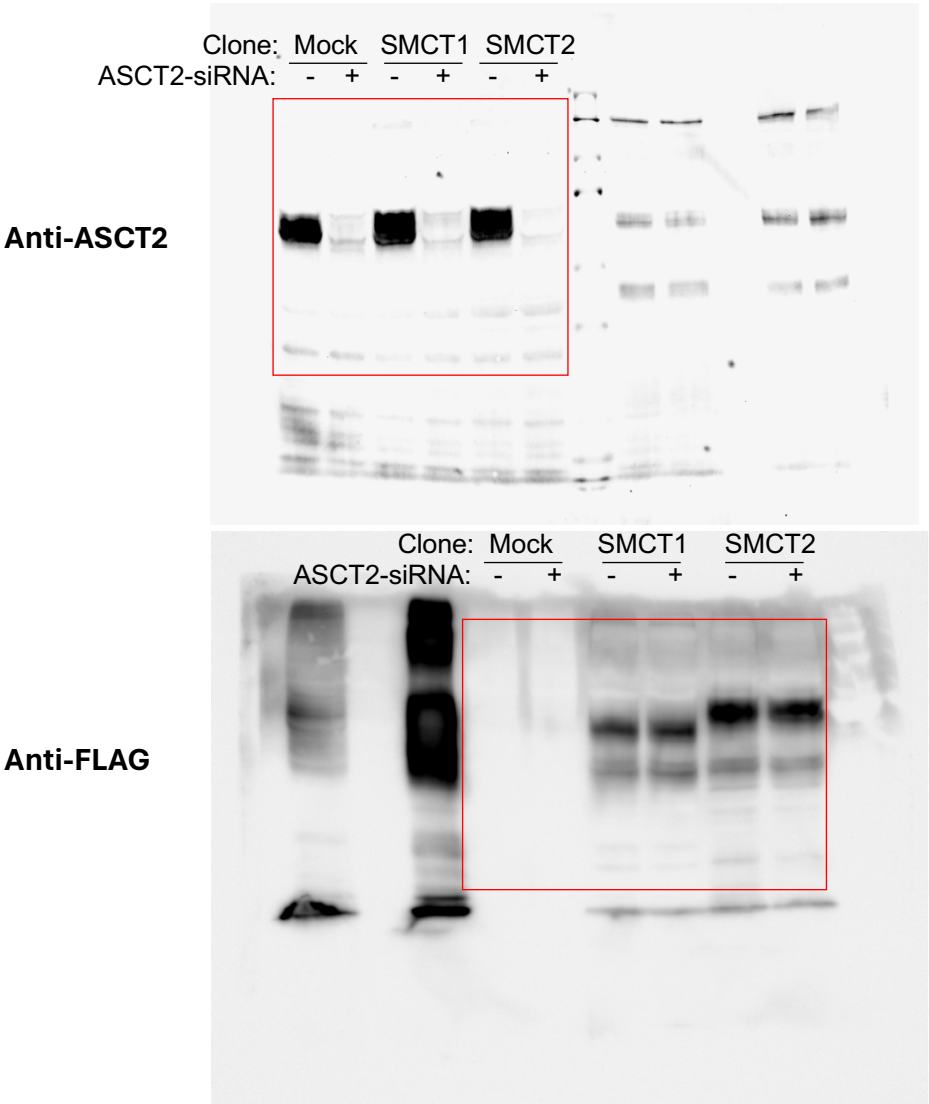

Supplement: Figure 5—figure supplement 1—source data 3. [file elife-92615-fig5-figsupp1-data3.pdf]

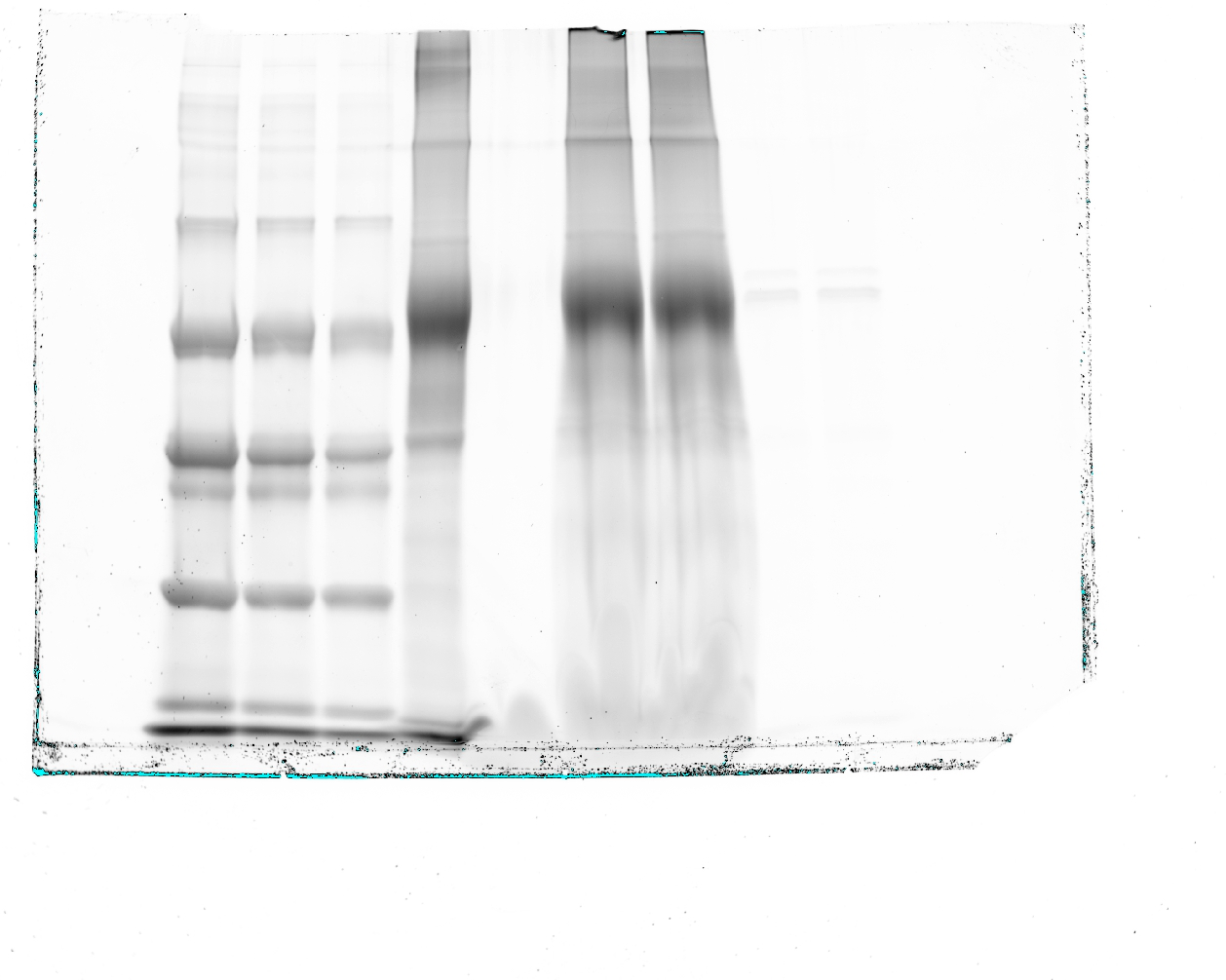

Supplement: Figure 6—source data 1. [file elife-92615-fig6-data1.zip › Figure 6 - souce data 1 (original gel).tif]

Figure 6

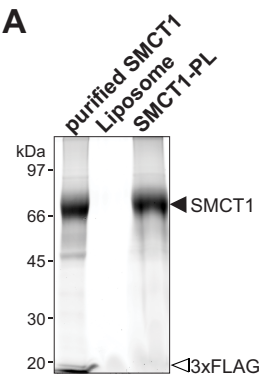

Source data

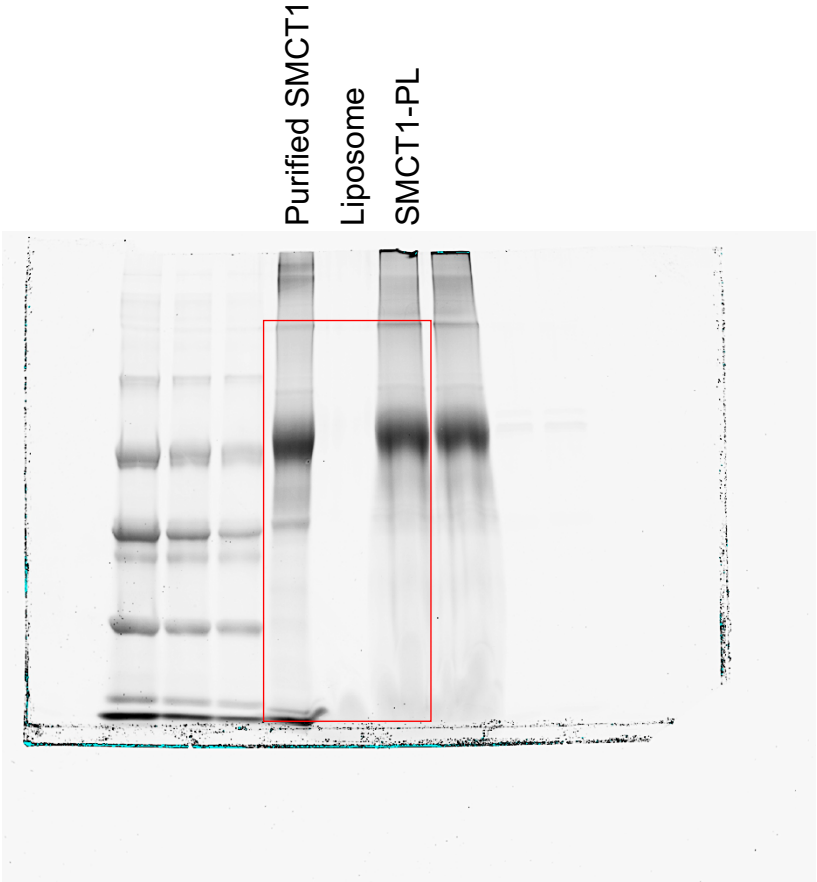

Supplement: Figure 6—source data 2. [file elife-92615-fig6-data2.pdf]
